# Electrochemical Corrosion and In Vitro Bioactivity of Nano-Grained Biomedical Ti-20Nb-13Zr Alloy in a Simulated Body Fluid

**DOI:** 10.3390/ma11010026

**Published:** 2017-12-27

**Authors:** Mohamed A. Hussein, Madhan Kumar, Robin Drew, Nasser Al-Aqeeli

**Affiliations:** 1 Center of Research Excellence in Corrosion, Research Institute, King Fahd University of Petroleum & Minerals (KFUPM), Dhahran 31261, Saudi Arabia; mahussein@kfupm.edu.sa (M.A.H.); madhankumar@kfupm.edu.sa (M.K.); 2 Department of Mechanical and Industrial Engineering, Concordia University, Montreal, QC H3G 1M8, Canada; robin.drew@concordia.ca; 3 Department of Mechanical Engineering, King Fahd University of Petroleum & Minerals (KFUPM), Dhahran 31261, Saudi Arabia

**Keywords:** biomaterial, electrochemical testing, polarization, Ti-Nb-Zr alloy, bioactivity permanent

## Abstract

The bioactivity and the corrosion protection for a novel nano-grained Ti-20Nb-13Zr at % alloy were examined in a simulated body fluid (SBF). The effect of the SPS’s temperature on the corrosion performance was investigated. The phases and microstructural details of the developed alloy were analyzed by XRD (X-ray Diffraction), SEM (Scanning Electron Microscopy), and TEM (Transmission Electron Microscope). The electrochemical study was investigated using linear potentiodynamic polarization and electrochemical impedance spectroscopy in a SBF, and the bioactivity was examined by immersing the developed alloy in a SBF for 3, 7, and 14 days. The morphology of the depositions after immersion was examined using SEM. Alloy surface analysis after immersion in the SBF was characterized by XPS (X-ray Photoelectron Spectroscopy). The results of the bioactivity test in SBF revealed the growth of a hydroxyapatite layer on the surface of the alloy. The analysis of XPS showed the formation of protective oxides of TiO_2_, Ti_2_O_3_, ZrO_2_, Nb_2_O_5_, and a Ca_3_(PO_4_)_2_ compound (precursor of hydroxyapatite) deposited on the alloy surface, indicating that the presented alloy can stimulate bone formation. The corrosion resistance increased by increasing the sintering temperature and the highest corrosion resistance was obtained at 1200 °C. The improved corrosion protection was found to be related to the alloy densification. The bioactivity and the corrosion resistance of the developed nanostructured alloy in a SBF renders the nanostructured Ti-20Nb-13Zr alloy a promising candidate as an implant material.

## 1. Introduction

The alloys to be developed for biomedical applications should have a high corrosion resistance, should be biocompatible, and should have suitable mechanical characteristics such as strength and modulus of elasticity [1]. The main metallic alloys developed for biomaterial applications include Ti alloys, stainless steels, and Co alloys [1,2,3,4]. Ti and Ti alloys possess corrosion protection, are biocompatible, and they offer a relatively low elastic modulus. Furthermore, these advantages result in a preference for Ti alloys in the medical and dentistry fields [5,6,7] and for prosthetic implants [8].

The development of Ti alloys with the addition of various constituents such as vanadium, nickel, chromium, and aluminum has been reported in the literature [9]. However, assessments usually extend beyond microstructural and mechanical characterizations to determine the corrosion protection, toxicity, and biocompatibility for use in the biomedical field. While V and Al (available in the widely used Ti-6Al-4V alloys) are not susceptible to corrosion, they likely have a toxic effect [5] and can cause mutagenic cytology and allergic reactions [6]. Ni shows a lower biocompatibility, while researchers have concerns regarding genotoxicity in Cr [9]. Therefore, the choice of suitable chemical components in a developed alloy for biomedical applications is a major concern.

Ti, Nb, and Zr have been reported as the non-toxic and the utmost compatible elements, which do not cause a negative tissue response [10]. The Ti-20Nb-13Zr alloys were developed for biomedical applications and have a favorable near-β structure [8]. In addition to good mechanical properties, an implant alloy should also exhibit good corrosion resistance [11,12]. Titanium-based alloys with Nb, Zr, Sn, or Ta have shown a high corrosion protection due to the development of passive layers [13,14]. The Ti-13Nb-13Zr alloy shows a greater resistance to corrosion, in comparison to the Ti-6Al-4V alloy [15].

Ti Nb Zr (TNZ) alloys are considered as the basic systems for hard tissue implant as they consist of non-toxic elements and have a high corrosion protection in human electrolytes [16]. The corrosion study of the Ti-13Nb-13Zr alloy has been investigated in the Ringer solution [13,17] and in Hank’s solution [18]. Ti alloys with a higher Nb content showed improvement in wear resistance and Nb_2_O_5_ showed good lubricating properties. Also, the Nb passivated quickly and its passive film remained for longer on the surface of the implant, with a higher Nb amount compared to the lower content of Nb [19,20]. Moreover, the higher Nb content contributed in the increase in the hardness of the Ti alloy [1]. Therefore, a new nano-grained alloy with a higher Nb content (Ti-20Nb-13Zr) at % has been developed by Hussein et al. [21] using mechanical alloying and Spark Plasma Sintering (SPS) methods. In their report, the details of the fabrication route, the microstructure and the microhardness were reported; however, the corrosion properties and the bioactivity of the developed alloy were not studied. Moreover, the influence of the sintering temperature on the performance of TNZ alloy has not been reported. Thus, the objective of the current work is to evaluate the bioactivity and the corrosion protection of the newly developed Ti-20Nb-13Zr at % alloy in a simulated body fluid (SBF), using linear potentiodynamic polarization and electrochemical impedance spectroscopy (EIS). The influence of the SPS temperature on electrochemical behavior was also investigated in detail. The layer formed after immersion in the SBF was studied using field emission scanning electron microscopy (FESEM) and XPS. 

## 2. Experimental Results and Discussion

### 2.1. Microstructure and Phase Constitutions

**Figure 1:**
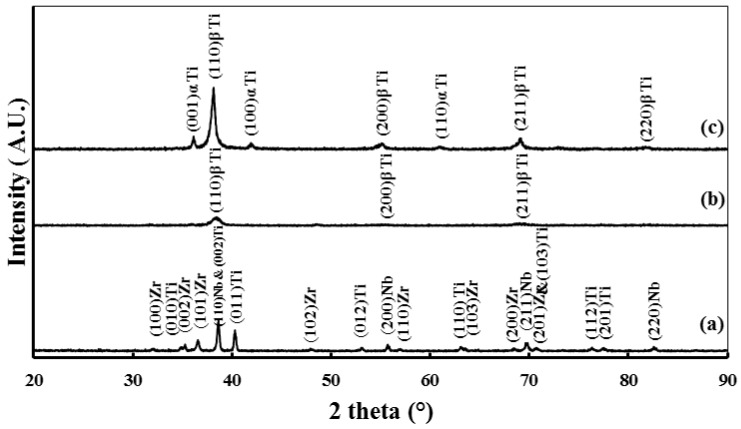
XRD patterns of Ti-20Nb-13Zr: (**a**) as-received powder mixture; (**b**) Blended elemental powder milled for 10 h; and (**c**) subjected to SPS at 1200 °C.

The influence of the SPS consolidation temperature on the microstructure investigations of the Ti-20Nb-13Zr alloy has been previously reported by the authors [21]. For the scope of the current work, we present XRD and FESEM results for the optimal SPS temperature of 1200 °C (temperature with the highest densification). Figure 1a depicts the XRD results of the raw blended elements in the Ti-20Nb-13Zr mixture, revealing all of the expected peaks for Ti, Nb, and Zr, and Figure 1b presents the XRD for the Ti-20Nb-13Zr alloy powder after 10 h of mechanical alloying (MA). The findings indicated a mixture of the β-Ti phase in a partially amorphous phase that formed as a result of a crystal defect evolution during the mechanical alloying process [22]. This result was confirmed by a broad peak at the position of the (110) β-Ti peak. After sintering at 1200 °C, the XRD pattern (Figure 1c) shows the coexistence of two phases: the β-Ti phase and the α-Ti phase. The percentage of the β-Ti phase is higher than that of the α-Ti phase, as indicated by the intensity of the peaks. 

From Ti alloy constitutions, the molybdenum equivalent (Mo Eq) has been used to identify the β-Ti phase stability as follows: Mo Eq = Mo + 0.67 V + 0.44 W + 0.28 Nb + 0.22 Ta + 1.6 Cr + 1.25 Ni + 1.7 Co + 2.9 Fe − 1.0 Al (wt %) [23]. For the developed alloy Ti-20Nb-13Zr at % alloy, the Mo Eq = 14.364. As reported in Ref. [24], a Mo Eq value above about 10.0 is required to stabilize β-Ti.

XRD pattern have been indexed and clearly showed the presence of the β-Ti phase (110), (200), (211), and (220). These results are in agreement with the previous report [25]. For the β-Ti phase, the lattice constant (a), has been calculated to form a 2 theta value for the β-Ti phase and it was 3.329 (Å). These results are in agreement with the previous report [26] for Ti-(13-20)Nb-(13-20)Zr wt % alloys with different compositions, in which the lattice constant of β-Ti phase ranged from 3.29 Å to 3.32 Å.

**Figure 2:**
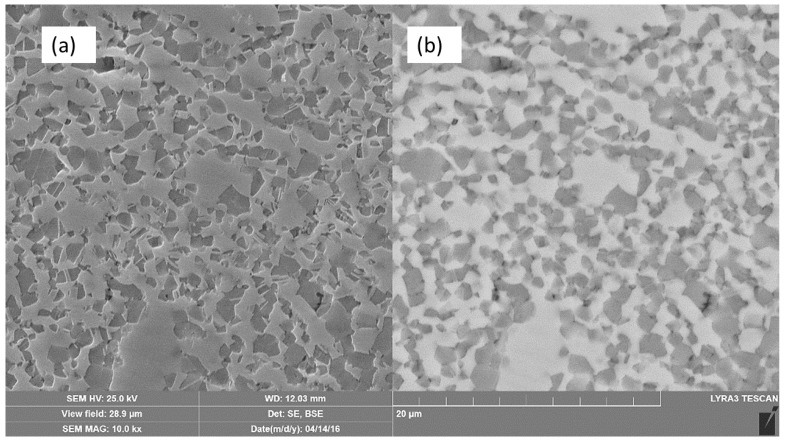
FESEM micrographs of the Ti-20Nb-13Zr alloy sintered at 1200 °C. (**a**) Secondary electron (SE); (**b**) Backscattered electron (BSE).

**Figure 3:**
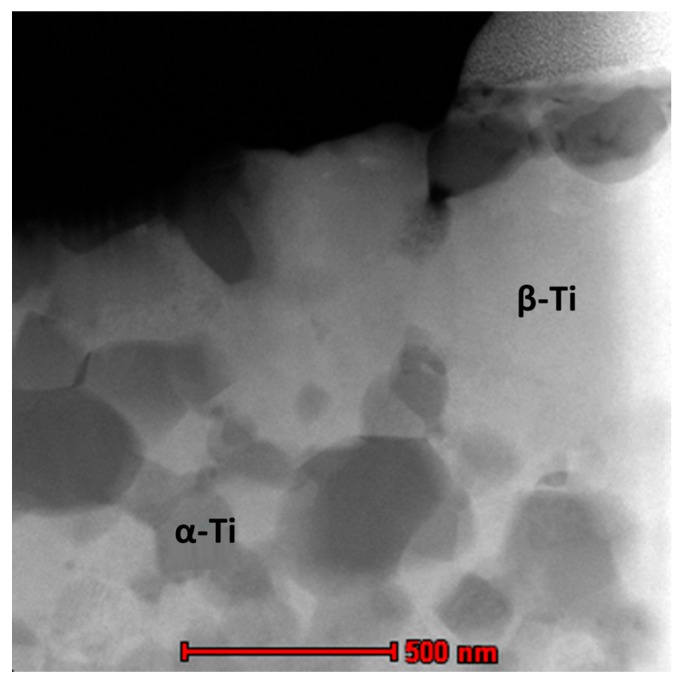
Bright-field TEM image for the alloy sintered at 1200 °C.

FESEM micrographs including the secondary electron (SE) and the backscattered electron (BSE) of the Ti-20Nb-13Zr alloy after SPS at 1200 °C are presented in Figure 2. It shows a β-Ti (bcc) matrix (whiter areas) surrounded by a α-Ti (hcp) region (darker areas), which confirms the XRD results. To confirm the chemical composition homogeneity, an EDX (Energy Dispersive X-ray Spectroscopy) attached to the SEM have been done on a different location in the BSE-FESEM image for both the β-Ti and the α-Ti phases. The results showed that the phase is chemically homogeneous and that the average composition in in β-Ti region was Ti-37.66Nb-18.3Zr (wt %), while the α-Ti phase showed the average composition of Ti-27.68Nb-18.9Zr (wt %). The reason that the Nb content was higher in the beta phase compared to the alpha phase was because the Nb is considered as a beta stabilizer, however, Zr was reported to be a stabilizer for both phases so that the Zr percentage is almost the same in both phases. The high resolution bright field TEM image of the alloy sintered at 1200 °C showing in Figure 3. The α-Ti grains with an average grain size of 127.6 nm were distributed in a matrix of β-Ti alpha.

### 2.2. Alloy Surface Analysis after Immersion in a SBF

**Figure 4:**
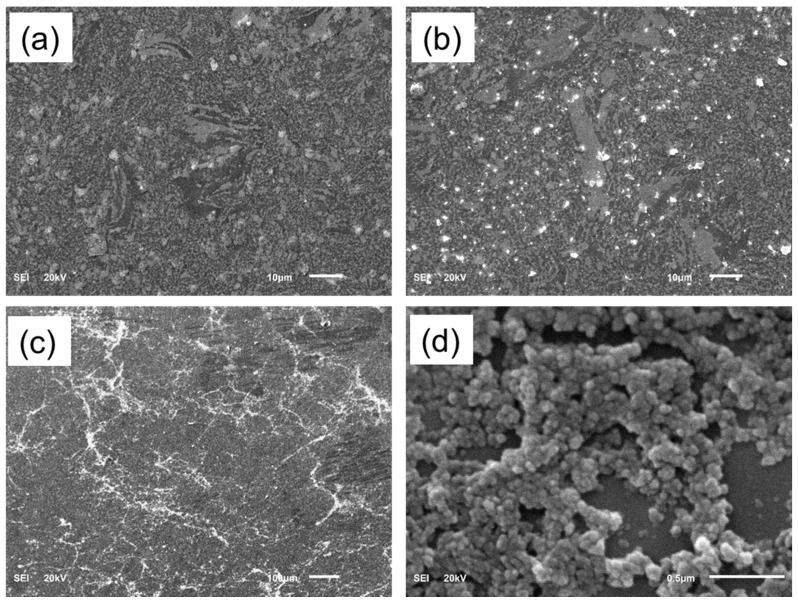
Surface morphology of the Ti-20Nb-13Zr alloy after immersion in the simulated body fluid (SBF) for different time (**a**) 3 days; (**b**) 7 days; (**c**) 14 days; (**d**) 14 days at a higher magnification.

**Figure 5:**
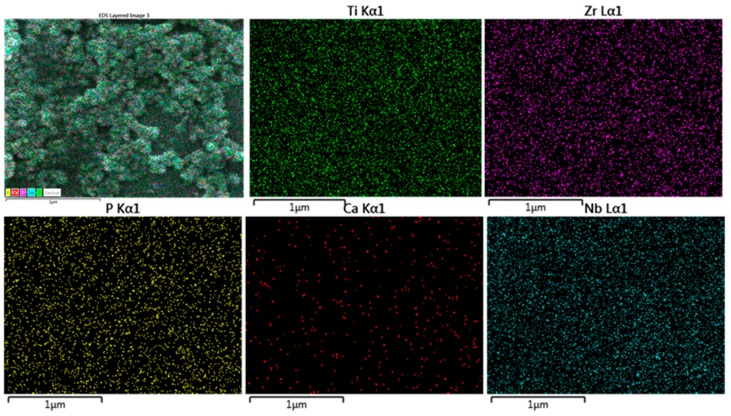
SEM-EDX mapping of surface morphology of the Ti-20Nb-13Zr alloy after immersion in the SBF for 14 days.

After SPS, in vitro bioactivity examinations were implemented in the SBF for selected samples sintered at 1200 °C, i.e., those showing the highest densification [21]. The in vitro bioactivity of the alloy was assessed by immersion of the alloy in the SBF for different times (3, 7, 14 days), then the surface morphology and precipitates were analyzed by SEM and XPS. After 3 days, there were scattered white particles deposited from the solution on the surface (Figure 4a) and this deposition grew by increasing the immersion time to 7 days (Figure 4b) and had covered most of the surface after 14 days of immersion (Figure 4c). The high magnification images of the sample immersed for 14 days showed nanoglobular particles covering the surface. Similar results were also obtained by Ref. [27]. The elemental mapping attached to the SEM was performed for the sample immersed for 14 days (Figure 5) and showed the existence of Ca and P, in addition to the alloying constitutions (Ti, Nb, Zr) of the alloy.

**Figure 6:**
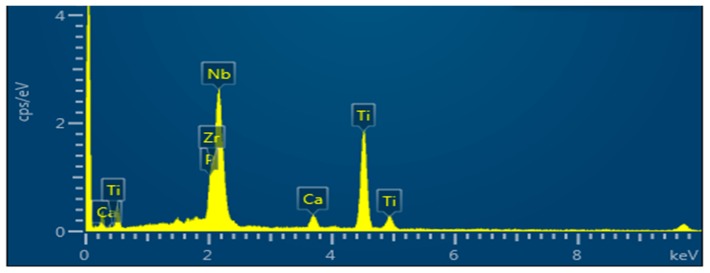
EDX of the sample surface after immersion in SBF.

Figure 6 depicts the EDX peaks for the sample surface after immersion in SBF. The presence of the main elements of apatite Ca, and P signals have been confirmed, along with the elements of alloy (Ti, Nb, Zr). The average ratio for Ca/P is 1.558 which is close to both the stoichiometric ratio in hydroxyapatite (HA) (1.67) [28], as well as the real ratio of 1.5 from the Ca_3_(PO_4_)_2_ compound [16].

**Figure 7:**
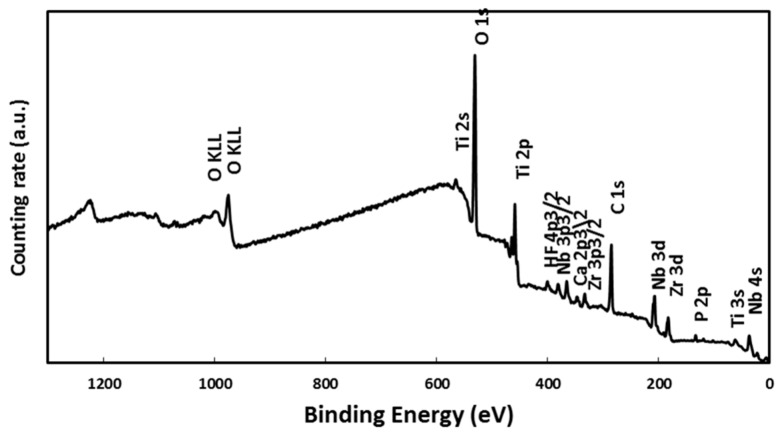
XPS survey spectrum of the Ti-20Nb-13Zr alloy surface after a 7-day immersion in a SBF.

For deep investigation about the constitutions of the deposited particles on the surface, an XPS investigation was carried out for the sample immersed for 7 days in SBF. The XPS survey spectrum presented at Figure 7 shows that the main components in the spectrum are: Ti 2p, Ti 2s, Nb 3d, Nb 4s, Nb 3p3/2, Zr 3d, Zr 3p3/2, P 2p, Ca 2p, C 1s, and O 1s [29,30,31]. The Ti 2p peak is indicative of TiO_2_; the Nb 3d, Nb 4s, and Nb 3p3/2 peaks correspond to the protective Nb_2_O_5_ oxide; the Zr 3d and Zr 3p3/2 peaks correspond to ZrO_2_ oxide; the P 2p peak indicates Ca_3_(PO_4_)_2_; and the Ca 2p peak corresponds to the presence of a Ca_3_(PO_4_)_2_ compound. An analysis of the XPS spectrum showed the existence of protective oxides of the alloy constitution elements (TiO_2_, ZrO_2_, and Nb_2_O_5_), which increases the corrosion protection, with carbon as a contaminant, in addition to calcium and phosphorous ions [32,33,34,35,36]. The deposition of a Ca_3_(PO_4_)_2_ compound (a precursor of hydroxyapatite) on the alloy surface indicates that the developed alloy can stimulate bone formation.

**Figure 8:**
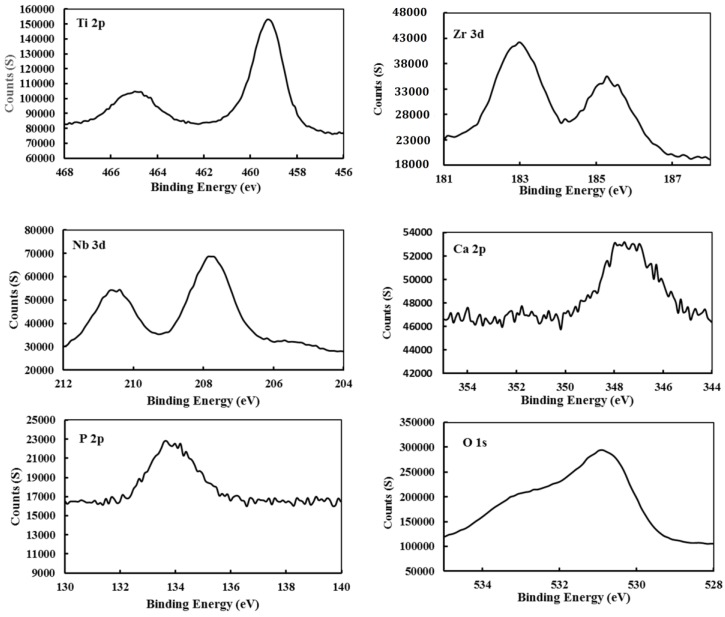
XPS spectra for Ti 2p, Nb 3d, Zr 3d, and P 2p, Ca 2p, and O 1s of the TNZ alloy sample surface after immersion in SBF.

Figure 8 shows the XPS spectra for Ti 2p, Nb 3d, Zr 3d, P 2p, Ca 2p, and O 1s of the TNZ alloy sample surface after immersion in SBF. The Ti 2p spectra reveal the double peaks for Ti 2P at 459 eV and at 464.8 eV which correspond to Ti_2_O_3_ and TiO_2_ [16]. The binding energy for Nb 3d showed double peaks at 207.6 eV and 210.4 eV which can be interpreted as Nb_2_O_5_ oxide [37,38]. Double peaks of Zr 3d at 183 eV and 185.2 eV confirm the presence of ZrO_2_ [38,39]. The spectra of P 2p shows the peak at a binding energy of 133.78 eV which indicated the presence of a Ca_3_(PO_4_)_2_ compound [40]. The Ca 2p peak proved the presence of the same Ca_3_(PO_4_)_2_ compound, as proved to form P 2p as well. The same trend of results was obtained by Ref. [16]. The spectrum of O 1s showed two peaks; the first one at 530.9 eV indicated the presence of oxides, while the second peak at 532.5 eV indicated the existence of water that was absorbed on the sample’s surface [40].

The mechanical stability of the oxide film of Ti alloy determines the behavior of the Ti alloy. For example, the common commercial Ti alloy forms Ti-6Al-4V systems. The formed TiO_2_ is stable, which results in improved corrosion protection. However, the V_2_O_5_ that began the dissolving process decreased the corrosion protection of the alloy. Therefore, the addition of metals like Nb and Zr reduce the release of metals because their oxides are less soluble in physiological fluids than oxides formed from aluminum and vanadium [40]. From literature, the TiNbZr alloy showed better corrosion resistance than commercial Ti, CP, and TiAlV [41,42], and Ti-Mo and TiAlNb alloys in physiological mediums [41].

### 2.3. Electrochemical Analysis

**Figure 9:**
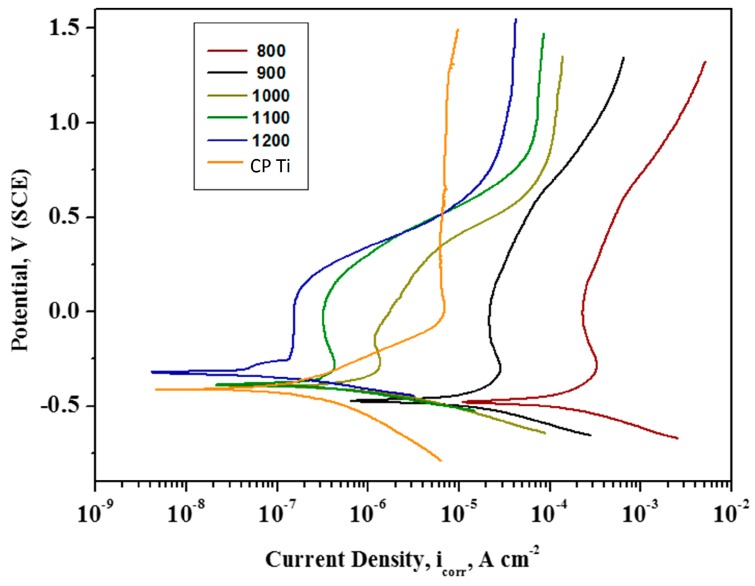
Potentiodynamic polarization curves for TNZ alloys subjected to SPS at different temperatures (temperature are in °C).

Figure 9 presents typical potentiodynamic polarization curves acquired for the Ti-20Nb-13Zr alloy substrates processed at different consolidation temperatures in a SBF medium. By closer observation of the anodic polarization branches for CP, Ti, and TNZ, there is indication of the typical active polarization starting from −300 mV to 100 mV, displaying a linear rise in the corrosion current density (i_corr_) along with the potential. Afterward, deviations began to show in the breakdown and repassivation with the potential arisen. Then the Ti surface changed to a completely passive region which was indicated by the constant current density [43]. It is clear that the nature of the anodic polarization branches remain identical for substrates with different sintering temperatures. However, the polarization curves move towards a more positive electrochemical direction, with increasing sintering temperature, signifying an improved corrosion resistance. In general, the i_corr_ value is anticipated to be low and E_corr_ value should be more positive for a noble corrosion-resistant surface [44]. The E_corr_ and i_corr_ values of the Ti-20Nb-13Zr alloy substrates were estimated by the Tafel extrapolation method; the obtained results are recorded in Table 1. From the table, it is clear that the Ti-20Nb-13Zr alloy substrates presented the nobler shift in E_corr_ value with an increasing sintering temperature. This behavior arises, owing to the production of a passivated layer that may act as a physical barrier for the dissolution of bulk metal, hence significantly decreasing the corrosion rate. In general, Ti and its alloys are deliberated as a kind of highly active material due to its moderately negative standard reduction potential on emf series (E_0_ = −1.63 V SHE). Where, emf is Electro motive force. Owing to their high reactivity, Ti and its alloys readily undergo oxidation when exposed to open environments and also in solutions, which results in the production of a protective/passive layer of Ti oxides due to a vigorous anodic reaction on the metallic surface. The passive films that were formed act as a physical barrier between the nearby surroundings and underneath the Ti surface, preventing the further corrosion of the Ti through the passive layer electrolyte interface. The existence of the protective/passive layer of oxides over the metallic surface is a forbidding obstacle to the corrosion phenomenon [45].

Furthermore, the i_corr_ values also decrease with an increasing sintering temperature. It has been previously reported that a metal substrate naturally becomes passivated when the critical anodic current density of an active–passive metal is lower than approximately 1.0 × 10^−4^ A/cm^2^ in an aerated medium [46]. Hence, the Ti-20Nb-13Zr alloy substrates processed above 1000 °C exhibited an enhanced passivation performance in the body fluid environment.

**Figure 10:**
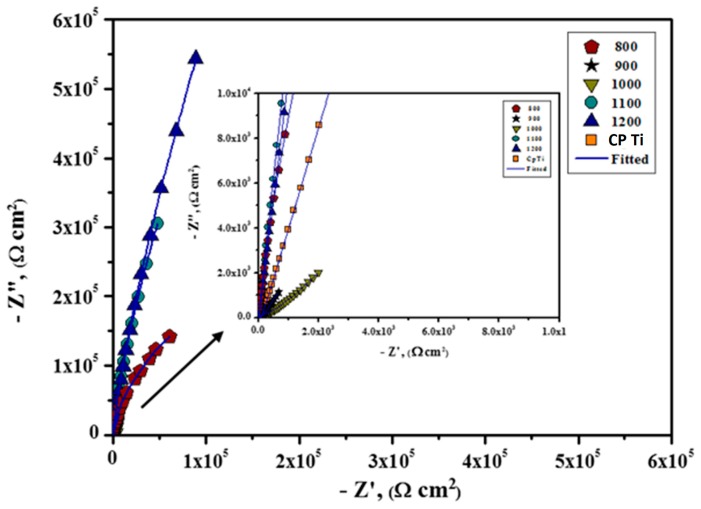
Nyquist plots of Ti-20Nb-13Zr alloys prepared at different sintering. Temperatures (°C) in a SBF.

EIS was performed to examine the electrochemical and passivation performance of the Ti-20Nb-13Zr alloy surface in the SBF medium and the obtained EIS data are displayed in both Bode and Nyquist formats. From the obtained Nyquist plots (Figure 10), it was noticeable that all of the investigated substrates exhibited a large partial capacitive arc and the length of the arc increased with the increasing sintering temperature during the same exposure period, revealing an improved electrochemical characteristic with increasing sintering temperature. Generally, the partial capacitive arc is ascribed to charge transfer reactions happening at the metal/solution interface or associated to the surface passive layer features [47]. By comparing the particle/incomplete capacitive arc of all of the Nyquist graphs, it was found that the corrosion resistance which is in reverse associated to corrosion rate follows the order: 1200 > CP Ti > 1100 > 1000 > 900 > 800 °C.

**Figure 11:**
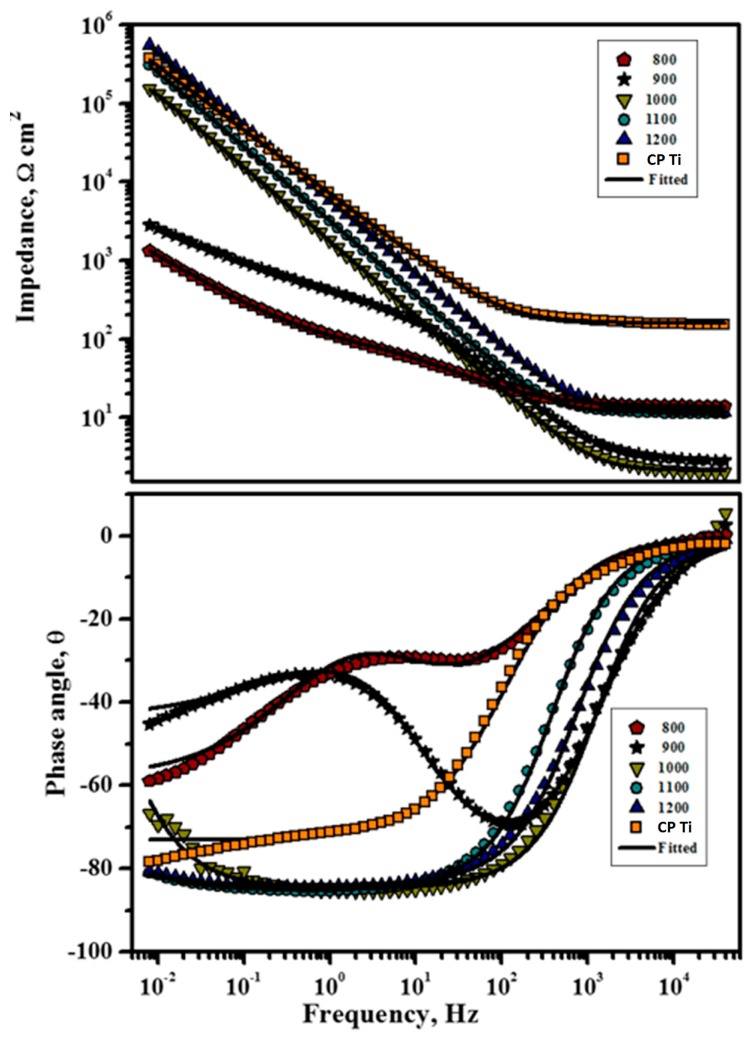
Bode plots of Ti-20Nb-13Zr alloys prepared at different sintering temperatures in a SBF: (**a**) Z magnitude plots and (**b**) phase angle plots.

**Figure 12:**
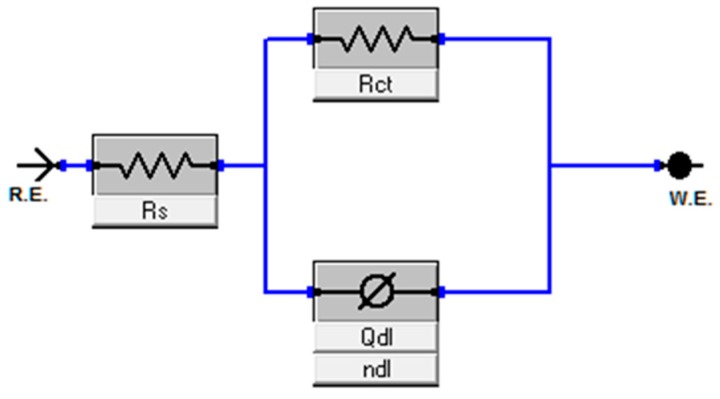
Equivalent electric circuit used to fit the experimental impedance data.

Only one time constant was obtained in both the Nyquist and Bode plots for all the Ti alloy substrates (Figure 10 and Figure 11), and the results can be adequately fitted by the Randles circuit presented in Figure 12.

The EIS parameters, R_s_ and R_ct_, denote the electrolytic and charge transfer resistance, respectively. Constant phase element (CPE/Q_dl_) was employed instead of non-ideal capacitance components owing to various physical aspects, including surface inhomogeneity resulting from surface roughness, dislocations or grain boundaries and impurities [48].

The impedance of a CPE can be written as

Z_CPE_ = [Y_0_ (j ω)^n^]^−1^ (1)

where Y_0_ and n represent the frequency of the independent factor and ω is angular frequency. The n value from zero to one is affected by the surface roughness. For the ideal surface, i.e., smooth surface, the n value reaches one and the impedance of CPE is considered as a pure capacitor [49].

Upon closer observation of the Bode resistance plots, it is clear that the impedance features in the high-frequency region can be absolutely estimated through its solution resistance. However, the Bode-phase plots display a plateau with phase angles close to −80° in the medium and low frequency regions, whereas the impedance value at low frequency increased linearly with the frequency, resulting in slopes close to −0.9, which is representative of passive films offering highly capacitive behavior [50,51]. In the low frequency region, high impedance values of approximately 105 Ω·cm^2^ were obtained for Ti-20Nb-13Zr alloy substrates above 1000 °C, while marginally lower values were observed for Ti-20Nb-13Zr alloy substrates processed at 800 and 900 °C. These results reveal a passive film with superior protective properties for alloys prepared at sintering temperatures above 1000 °C. Table 2 summarizes the EIS parameters calculated from the equivalent circuit that was fit to the EIS spectra for Ti-20Nb-13Zr alloy substrates in the SBF medium. 

From Figure 12 and Table 2, the high value of R_ct_ implies an enhanced corrosion resistance and the surface roughness factor, n for all samples that exhibit attaining a perfect capacitor which is close to 1. The sign of strong capacitance features is a linear slope of impedance and a phase angle close to 90° in the Bode plots. In general, R_s_ was pointedly unaltered by Ti substrates, however the charge transfer resistance (R_ct_) of the TNZ surface increased in the following order: 800 < 900 < 1000 < 1100 < CP Ti < 1200 °C. It has been already established that the high density of grain boundaries in metals with nano-size grains improves the production of a passive layer on the metallic surface [52], which subsequently enhances the corrosion resistance through restraint of the interaction between metals in an aggressive situation. In general, a thicker passive layer could be much more impervious in an aggressive environment. Furthermore, the natural oxide layer on the metallic substrates has the high density owing to the high grain boundary’s density [53]. Accordingly, the denser passive layer of nanostructured TNZ with an increasing sintering temperature led to an enhancement of the corrosion resistant behavior of the Ti substrates. 

Furthermore, the capacitance of the passive film is also most accountable for the corrosion resistant behavior of Ti alloy. In general, the capacitance, C and the thickness, d are inversely associated by the relation, C = ε·ε_0_·A/d, where ε represents the dielectric constant of the barrier, ε_0_ represents the vacuum permittivity, A represents the area, and d represents the thickness of the oxide layer [54]. Hence, the higher the capacitance of the material, the lower the thickness of the passive layer will be. The passive layer capacitance for the TNZ samples increased with the increasing sintering temperature. Among all the alloy substrates under investigation, the capacitance value is least for the TNZ substrates prepared at 1200 °C and the thickness of the barrier layer will be much higher than that of other samples. Furthermore, by comparing the heterogeneous factor (n_dl_), it obviously revealed that TNZ in higher sintering temperatures exhibited the highest n_dl_, which is close to 1 (ideal capacitor) designating that the formed passive layers are highly insulating and resistant to corrosion [55]. Whereas, TNZ with a lower sintering temperature displayed the lowest n_dl_ values, which suggests that it owns some defects, therefore permitting aggressive species to find solutions and creating susceptibility to corrosion on Ti. Hence, the higher R_ct_ and the lower Q_dl_ values observed with an increasing sintering temperature indicate a nobler electrochemical performance, which is in good agreement with the potentiodynamic polarization results. 

From the electrochemical investigations, it could be concluded that the corrosion resistance behavior of the Ti-20Nb-13Zr alloy substrates increased with sintering temperature. Density calculations showed that the density increased by increasing the sintering temperature which can be mainly attributed to high temperature diffusion. The densification was 96.7% at a sintering temperature of 800 °C, and 98.3% at 900 °C and increased linearly to 99.53% at 1200 °C.

It has already been studied that Ti-24Nb-4Zr-7.9Sn alloy samples sintered at low temperatures possess low density and are porous in nature, and then exhibit a lower corrosion protection, while the alloy consolidated at high temperatures show high densifications and are less porous in nature, displaying the enhanced corrosion protection. Hence, in the present investigation, the samples prepared above 1000 exhibited the highest corrosion resistance performance amongst others, which is ascribed to the increase of the Ti-20Nb-13Zr alloy’s density and less porous nature over alloy surfaces under these conditions [55].

## 3. Materials and Experimental Work

### 3.1. Synthesis of Alloy

Ti-20Nb-13Zr at % mixture was prepared from elemental powders of Ti, Nb, and Zr (of −325 mesh size and 99.8% purity), with 13% Zr and 20% Nb and (remainder Ti) blended at an atomic percentage. The powders were subjected to mechanical alloying in a planetary ball and were then sintered using a SPS instrument (Type HP D-5, Fine Ceramics Technologies, Rauenstein, Germany) at different temperatures (800, 900, 1000, 1100, and 1200 °C). The SPS pressure was kept constant at 50 MPa, a 100 °C/min heating rate was used, and the samples were held at the consolidation temperature for 10 min. The developed phases of the milled alloy powders and the consolidated alloy were characterized by XRD (AXSD8, Bruker, Germany), and Cu-Kα radiation (scanning speed of 1 degree/min). FESEM (Tescan Lyra-3, TESCAN, Brno, Czech Republic) was used to examine the microstructure of sintered alloy and after its immersion in SBF. TEM (Tecnai G Series, USA operated at 200 keV) have been used to investigate the microstructure. After sintering, the alloy density was measured experimentally using a density kit (METTLER Toledo, Zürich, Switzerland) by measuring the density of the sintered alloy in the air and on water [56]. The reported value was the average of three measurements. Then the densification was calculated using the relation; Densification (%) = (experimental density/theoretical density) × 100.

### 3.2. In Vitro Bioactivity Test in a SBF

The bioactivity was evaluated by immersing the sample in a SBF for 3, 7, 14 days to evaluate its ability to favor apatite deposition. The SBF was prepared and the in vitro characterization was performed as reported in [57] to prepare the SBF (pH 7.4, 1 L), with an ionic concentration nearly equal to that of human body plasma [57]. Then the sample was carefully washed with water (distilled) and dried prior to SEM investigation.

### 3.3. Surface Analysis of the Alloy by XPS

Subsequently, the morphology of the depositions after immersion in SBF were characterized using a SEM (Japan Electron Optics Laboratory Company, Tokyo, Japan). The constitution of the film deposited on the surface of the alloy was examined by XPS. The XPS apparatus (ESCALAB 250 XI, Thermo Scientific, Waltham, MA, USA) had a base pressure of 6.12 × 10^−10^ mbar in the chamber. The X-ray source was Al Kα radiation from an anode operating at 130 W with a spot size of 650 nm; an energy step size of 1 eV was used. 

### 3.4. Electrochemical Analysis

The electrochemical analysis was implemented using potentiodynamic polarization techniques and EIS experiments in a SBF solution to examine the corrosion performance of the new Ti-20Nb-13Zr alloys. All analyses were performed in a conventional three-electrode assembly with the newly developed Ti-20Nb-13Zr alloy substrates as the working electrode with the exposed area of 1.0 cm^2^. A graphite rod and a saturated calomel electrode (SCE) served as the auxiliary and the reference electrode, respectively. The electrochemical measurements were initiated after approximately 30 min of substrate immersion in the SBF medium to reach a constant open circuit potential (OCP) value. A scan range from 250 mV below the OCP to 1500 mV was selected for the potentiodynamic polarization analysis, with a scanning rate of 0.197 mV/s. The corrosion rate was estimated by the Tafel extrapolation method. 

EIS spectra were collected in the frequency range of 100 kHz to 10 mHz with a 10-mV-amplitude AC signal. The impedance diagrams were displayed in the Bode plot representation. All the electrochemical studies were repeated at least three times.

## 4. Conclusions

The influence of the SPS temperature on corrosion resistance was investigated in a SBF for the newly developed near-β-type Ti-20Nb-13Zr processed by MA and SPS methods for the first time. The bioactivity was also studied by immersion in a SBF for 3, 7, 14 days. The following points summarize the obtained results.

1. Structure and microstructure analysis confirmed the formation of the nano-grained alpha phase.
2. An analysis of the XPS spectra revealed the existence of protective oxides of the alloy (TiO_2_, ZrO_2_ and Nb_2_O_5_), which enhance the corrosion protection of the presented alloy.
3. The deposition of a Ca_3_(PO_4_)_2_ compound (a precursor of hydroxyapatite) on the surface of the developed alloy indicates that the presented alloy can stimulate bone formation.
4. From electrochemical corrosion studies, it was concluded that the newly developed Ti-20Nb-13Zr alloy substrates exhibited higher corrosion protection for sintering temperatures at 1200 °C.
5. The bioactivity and corrosion resistance of the developed nanostructured alloy in a SBF medium renders the nanostructured Ti-20Nb-13Zr alloy a promising candidate as an implant material.

## Figures and Tables

**Table 1:** Tafel parameters for CP Ti and alloys consolidated at different temperatures.

Sintered Temperature (°C)	E_corr_ (mV)	i_corr_ (µA cm^2^)	β_a_ (mV/dec)	β_b_ (mV/dec)	Corr. Rate (mpy) × 10^−3^
CP Ti	−418	50.90	84	69	44.90
800	−478	149.285	94	79	63.840
900	−474	18.232	65	77	8.336
1000	−397	1.240	94	74	0.565
1100	−388	0.438	85	69	0.200
1200	−328	0.060	72	83	0.0275

**Table 2:** EIS results.

Sintered Temperature (°C)	R_s_ (cm^2^)	R_ct_ (kΩ·cm^2^)	Q_dl_ (µA·cm^2^)	N
CP Ti	25.98	397	41.82	93.27
800	12.73	1.226	3879	92.49
900	11.98	2.837	624.71	91.53
1000	15.36	154	93.72	94.32
1100	14.86	306	54.44	94.67
1200	15.32	530	30.03	95.12
